# Characterization of Emerging Serotype 19A Pneumococcal Strains in Invasive Disease and Carriage, Belgium

**DOI:** 10.3201/eid2808.212440

**Published:** 2022-08

**Authors:** Stefanie Desmet, Heidi Theeten, Lies Laenen, Lize Cuypers, Piet Maes, Wouter Bossuyt, Liesbet Van Heirstraeten, Willy E. Peetermans, Katrien Lagrou

**Affiliations:** University Hospitals Leuven, Leuven, Belgium (S. Desmet, L. Laenen, L. Cuypers, W. Bossuyt, W.E. Peetermans, K. Lagrou);; Katholieke Universiteit Leuven, Leuven (S. Desmet, P. Maes, W. Bossuyt, W.E. Peetermans, K. Lagrou);; University of Antwerp, Antwerp, Belgium (H. Theeten, L. Van Heirstraeten);; Katholieke Universiteit Leuven‒University Hospitals Leuven, Leuven (W. Bossuyt)

**Keywords:** pneumococcal strains, bacteria, vaccines, serotypes, emerging serotype 19A, invasive disease, carriage, respiratory infections, vaccine-preventable diseases, Belgium, Streptococcus pneumoniae, antimicrobial resistance

## Abstract

After switching from 13-valent to 10-valent pneumococcal conjugate vaccine (PCV10) (2015–2016) for children in Belgium, we observed rapid reemergence of serotype 19A invasive pneumococcal disease (IPD). Whole-genome sequencing of 166 serotype 19A IPD isolates from children (n = 54) and older adults (n = 56) and carriage isolates from healthy children (n = 56) collected after the vaccine switch (2017–2018) showed 24 sequence types (STs). ST416 (global pneumococcal sequence cluster [GPSC] 4) and ST994 (GPSC146) accounted for 75.9% of IPD strains from children and 65.7% of IPD (children and older adults) and carriage isolates in the PCV10 period (2017–2018). These STs differed from predominant 19A IPD STs after introduction of PCV7 (2011) in Belgium (ST193 [GPSC11] and ST276 [GPSC10]), which indicates that prediction of emerging strains cannot be based solely on historical emerging strains. Despite their susceptible antimicrobial drug profiles, these clones spread in carriage and IPD during PCV10 use.

Pneumococcal serotype 19A is one of the 100 known serotypes of *Streptococcus pneumoniae* (*1*). The high potential of serotype 19A to cause invasive pneumococcal disease (IPD), its high rates of antimicrobial drug resistance, the variable inclusion of this serotype in conjugate vaccines, and its high genetic plasticity makes it one of the most studied pneumococcal serotypes (*2*–*4*). Serotype 19A became more prevalent after 7-valent pneumococcal conjugate vaccine (PCV7) was introduced into childhood vaccination programs. As a consequence of the decrease of PCV7 serotype IPD, serotypes not included in PCV7, such as serotype 19A, became a more critical issue because of process called serotype replacement. In the United States, capsular switching and antimicrobial drug resistance played a major role in the increase of serotype 19A after introduction of PCV7 (*2*,*3*).

In Belgium, introduction of the 13-valent vaccine, which includes serotype 19A, resulted in a 10-fold decrease of serotype 19A incidence in the youngest children (*5*). Likewise in Belgium, in most countries that use PCV13, a decrease in serotype 19A IPD in children was observed, resulting in a low residual serotype 19A IPD incidence during PCV13 use (*6*,*7*). By an indirect effect, use of PCV13 in children also resulted in a decrease in the incidence of serotype 19A IPD in older adults in Belgium and other countries (*7*,*8*). However, serotype 19A has remained one of the major serotypes, accounting for 5.6% of IPD cases in older adults during 2015 (*9*). In addition, in other countries (e.g., England and Wales), serotype 19A did not completely disappear after PCV13 introduction, and the number of serotype 19A IPD cases remained on a plateau (*10*).

For >20 years, a stable national laboratory-based surveillance of IPD has been in place in Belgium (*5*). Since 2016, a national nasopharyngeal carriage study investigating pneumococcal carriage in children attending day care centers was also conducted in parallel with IPD surveillance (*11*). These 2 parallel surveillances make it possible to study in detail changes over time in the pneumococcal population (*4*,*5*,*11*). After a decrease in pediatric IPD incidence after the switch from PCV7 to PCV13 in Belgium, a major increase in IPD incidence in the youngest children was again observed during 2017–2018, two years after the switch from PCV13 to PCV10 (2015–2016) (*5*). This increase was attributed mainly to the major increase in serotype 19A IPD (from 2.2 cases/100,000 children <2 years old in the PCV13 period to 12.5 cases/100,000 children <2 years old in PCV10 period) (*5*). In older adults, an increase in the proportion of serotype 19A IPD was detected from 5.6% during 2015 to 13.2% during 2019 (*9*). In parallel, an increase in the proportion of serotype 19A in nasopharyngeal carriage of young children in Belgium was observed from 0.4% during 2016 to 6.4% during 2017–2018 (*11*).

To elucidate the rapid increase in serotype 19A IPD and carriage, we performed phenotypic and molecular characterization of the serotype 19A strains isolated during 2017–2018. We investigated whether the same serotype 19A strains were detected in children and adults who had IPD, and if they correspond to the ones carried by the youngest children during 2017‒2018. Moreover, we aimed to compare these serotype 19A strains from Belgium with serotype 19A IPD strains that were detected after introduction of PCV7 in Belgium and other countries to investigate whether there was a clonal expansion of previous dominant clones and whether capsular switching or antimicrobial drug resistance played a role in reemergence of serotype 19A.

## Materials and Methods

### Bacterial Strains

We included in the study all serotype 19A *S. pneumoniae* isolates sent to the Belgian Reference Centre for Invasive *S. pneumoniae* and collected from a normally sterile site (e.g., blood culture, cerebrospinal fluid, pleural fluid, or synovial fluid) during 2010, during 2012–2018 from children <2 years of age (IPD children), and during 2018 from older adults (65–85 years of age) (IPD older adults). Data collection was part of the national passive surveillance network that showed a mean representativeness for IPD in Belgium of 90.5% (2007–2018) (*5*).

We collected serotype 19A pneumococcal strains carried by young healthy children 6‒30 months of age during a national nasopharyngeal carriage study (carriage children). This study has been described in detail (*12*). Healthy children were recruited in randomly selected daycare centers in 3 regions of Belgium (Flanders, Wallonia, and Brussels). We obtained a nasopharyngeal flocked swab specimen from 1,855 children during the winter of years 2 (2016–2017) and years 3 (2017–2018). For this study, we included only serotype 19A strains collected during 2017 and 2018. We defined 2010 as the PCV7 year, January 2012‒December 2014 as the PCV13 period, and January 2017‒December 2018 as the PCV10 period.

### Phenotypic Characterization

We performed serotyping of pneumococcal strains by detection of the Quellung reaction using serotype-specific antisera (SSI Diagnostica, https://ssidiagnostica.com). We conducted antimicrobial susceptibility testing by using disk diffusion for penicillin (oxacillin), erythromycin, tetracycline, trimethoprim/sulfamethoxazole, and levofloxacin. If the oxacillin zone diameter was <20 mm, we determined the MIC by using Etest (bioMérieux, https://www.biomerieux.com) for penicillin and cefotaxime. We interpreted results by using the European Committee on Antimicrobial Susceptibility Testing (https://www.eucast.org) 2019 guidelines. We interpreted a penicillin MIC >0.064 mg/L and a cefotaxime MIC >0.5 mg/L as indicating resistance.

### Genotypic Characterization

We extracted DNA by using the DSP DNA Mini Kit on the QIAsymphony SP/AP Instrument (QIAGEN, https://www.qiagen.com) and the protocol for gram-negative bacteria. We prepared libraries by using the Nextera XT DNA Library Prep Kit (Illumina, https://www.illumina.com) and the Echo 525 Liquid Handler Instrument (Beckman-Coulter, https://www.beckmancoulter.com), followed by solid-phase reversible immobilization bead purification (Hamilton, https://www.hamiltoncompany.com). 

We used a genomic DNA concentration of 0.2 ng/μL dissolved in 500 nL of nuclease-free water. We prepared a Nextera library according to the manufacturer’s (Illumina) protocol with a tagment DNA buffer to amplicon tagment mixture ratio of 2:1 and tagmentation time of 15 min at 55°C. We performed amplification of the library by using 12 PCR cycles of 95°C for 10 s, 55°C for 30 s, and 72°C for 30 s. 

We purified beads by using Hamilton NGS Star and AMPure XP beads (Beckman-Coulter) at a ratio of 30%. We verified library quality by using Bioanalyzer (Agilent, https://www.agilent.com) and a quantitative PCR (Kapa Biosystems, https://kapabiosystems.com). We performed sequencing by using the MiSeq System with MiSeq version 3 PE300 reagents and Hiseq2500 with version 2 reagents (Illumina). We submitted read data for all *S. pneumoniae* serotype 19 isolates to the National Center for Biotechnology Information Short Read Archive (BioProject accession no. PRJNA780376).

We analyzed demultiplexed sequence reads by using 2 pipelines. First, we used an in-house cloud-based pipeline based on Kraken 2, the Centers for Disease Control and Prevention StrepLab pipeline, and SeroBA (*13*,*14*). We used Kraken 2 and information about identification of the strain to check whether there was contamination with other bacterial species (*15*). Combining of the Centers for Disease Control and Prevention StrepLab pipeline and SeroBA resulted in an output of serotype, multilocus sequence typing (MLST) results, presence of pilus genes, presence of antimicrobial drug resistance genes, assignment of penicillin-binding protein (PBP) profile, and prediction of antimicrobial drug susceptibility of the strain.

Second, we used the INNUca pipeline (https://innuca-nf.readthedocs.io) for performing quality control and de novo assembly of the genome. We performed quality control of reads by using FastQC version 0.11.5 (https://guix.gnu.org), and cleaned and trimmed reads by using Trimmomatic version 0.36 (https://kbase.us/applist/apps/kb_trimmomatic/run_trimmomatic/release).

We assembled the genome by using SPAdes version 3.11.0 (https://cab.spbu.ru) and subsequently polished the genome by using Pilon version 1.18 (https://github.com/broadinstitute/pilon). We used the assembled genome for assignment of global pneumococcal sequence clustering (GPSC) by using Pathogenwatch (Welcome Sanger Institute, https://pathogen.watch) (*16*). We used PopPUNK 2.4 (https://poppunk.readthedocs.io) and a newer GPSC reference database (n = 42,000) for strains that had a novel GPSC assignment by Pathogenwatch (accessed on January 11, 2021).

### Data Analysis

We compared phenotypic and genotypic characteristics of serotype 19A strains causing IPD in older adults and carried (isolated during 2017–2018) by healthy young children with serotype 19A IPD strains isolated from children during the PCV7 period (2010), the PCV13 period (2012–2014), and the PCV10 period (2017–2018). We compiled descriptive statistics for these comparisons.

## Results

A total of 255 serotype 19A strains were included in this study, of which 166 strains were isolated in the PCV10 period (2017–2018): 54 IPD strains from children, 56 IPD strains from older adults, and 56 carriage strains from children. A total of 89 strains that were isolated in children <2 years old in the PCV7 (2010) (n = 67) and PCV13 period (2012–2014) (n = 23) were included to analyze the evolution over time of the serotype 19A strains in children <2 years of age.

### Antimicrobial Drug Susceptibility and Pilus Genes of Serotype 19A Strains Isolated during 2017–2018

Resistance rates for serotype 19A strains were 9.0% for penicillin, 23.5% for erythromycin, 0.0% for levofloxacin, and 22.3% for tetracycline. Higher resistance rates for penicillin were detected in IPD strains (13.0% for children and 11.1% for older adults) compared with carriage strains (5.4%). For erythromycin and tetracycline, higher resistance rates were observed in IPD strains from children (29.6% and 27.8%) compared with IPD strains from older adults (17.9% and 17.9%) and carriage strains (23.2% and 21.4%) (Table 1). The pilus 1 gene was found in 47.6% of the serotype 19A strains in the PCV10 period, with a similar proportion in the different groups (range 46.3%–48.2% pilus-1‒positive strains in each group). Both pilus 1 and pilus 2 genes were detected in only 3 strains causing IPD in young children, 2 strains causing IPD in older adults, and 4 strains carried by young children in the PCV10 period. A total of 77 (46.4%) of the 166 serotype 19A strains had no pilus genes.

**Table 1:** Characteristics and antimicrobial drug susceptibility for serotype 19A strains of pneumococci isolated during the period after the PCV13 to PCV10 switch, Belgium*

Characteristic	IPD, young children	IPD, older persons	Carriage, young children	Total
No. strains	54	56	56	166
Year of isolation	2017–2018	2018	2017–2018	2017–2018
Age				
0–11 mo	41	0	8	49
13–23 mo	13	0	32	45
2–3 y	0	0	15	15
3–4 y	0	0	1	1
65–85 y	0	56	0	56
Sex, M/F	32/22	27/29	28/28	87
Source of isolation				
Blood	48	56	0	104
CSF	5	0	0	5
Pleural fluid	1	0	0	1
Nasopharyngeal swab specimen	0	0	56	56
Penicillin resistant	7 (13.0)	5 (11.1)	3 (5.4)	15 (9.0)
Levofloxacin resistant	0 (0)	0 (0)	0 (0)	0 (0)
Erythromycin resistant	16 (29.6)	10 (17.9)	13 (23.2)	39 (23.5)
Tetracycline resistant	15 (27.8)	10 (17.9)	12 (21.4)	37 (22.3)
Pilus 1	25 (46.3)	27 (48.2)	27 (48.2)	79 (47.6)
Pilus 1 and pilus 2	3 (5.6)	2 (3.6)	4 (7.1)	9 (5.4)

*Values are no. or no. (%). CSF, cerebrospinal fluid; IPD, invasive pneumococcal disease; PCV, pneumococcal conjugate vaccine.

### MLST Types of Serotype 19A Isolated during 2017–2018

We detected 24 MLST types. ST416 (47.6%, 79/166), ST994 (18.1%, 30/166), ST2081 (5.4%, 9/166), ST320 (3.0%, 5/166), and ST419 (3.0%, 5/166) were the predominant types (Table 2). We detected 11 STs only once: ST63, ST1756, ST2013, ST2669, ST2927, ST3012, SST9387, ST13097, ST13701, ST16628, and ST16627. ST16628 and ST16627 were assigned as new pneumococcal STs. Of the predominant STs, we detected ST416, ST994, and ST320 in all 3 groups, and these STs accounted for >60% of the strains in each group: 43/54 (79.6%) for IPD young children, 36/56 (64.3%) for IPD older adults, and 35/56 (62.5%) for carriage strains. We detected ST416 in all groups, and it was the most predominant ST, accounting for 45%–48% of the strains in each group. ST2081, ST419 and ST1848 accounted for 26.8% (15/56) of carriage strains but were not detected as the cause of IPD in young children and only rarely (3.6%, 2/56) in older adults who had IPD. We detected 5 STs in children who had IPD, but we did not detect those STs in carriage. We detected ST994 at a higher proportion in IPD (15/54; 27.8%) than in carriage (6/56, 10.7%) in children.

**Table 2:** Characteristics of serotype 19A pneumococcal sequence types after the PCV13 to PCV10 switch, Belgium*

GPSC	ST	No.	IPD in person <2 years of age	IPD in person >65 years of age	Carriage in children <3 years of age	Pilus 1	No pilus	Penicillin resistant	Most frequent PBP profile	Erythromycin resistant	Resistance gene	Tetracycline resistant	Resistance gene
GPSC4	ST416	79	26	26	27	76	3	0	0-0-0 (74); new-0-0 (3); 1-0-0 (2)	17	*erm*(B)	15	*tetM*
	ST2081	9	0	1	8	0	9	0	1-0-187	0	ND	0	ND
	ST419	5	0	1	4	0	5	0	1-0-187	0	ND	0	ND
	ST199	4	0	4	0	0	4	0	1-0-187	0	ND	0	ND
	ST3017	3	1	2	0	0	3	0	1-0-187	0	ND	0	ND
	ST15414	2	1	1	0	2	0	0	0-0-0	0	ND	0	ND
GPSC146	ST994	30	15	9	6	0	30	0	2-4-0 (29); new-4-0 (1)	0	ND	0	ND
GPSC1	ST320	5	2	1	2	0	5	5	13-11-16	5	*erm*(B), *mefA*	5	*tetM*
GPSC11	ST193	4	1	0	3	0	4	0	2-0-2 (2); 2-0-3 (1)	4	*erm*(B)	3	*tetM*
GPSC10	ST276	4	0	4	0	0	4	4	17-39-18	4	*erm*(B)	4	ND
GPSC109	ST13096	4	2	2	0	0	4	0	1-4-3	0	ND	0	ND
GPSC99	ST1848	3	0	0	3	0	3	0	New-44-0 (2); 2-44-2 (1)	3	*erm*(B)	3	*tetM*
GPSC10	ST15077	2	2	0	0	0	2	2	17-89-18	2	*erm*(B)	2	*tetM*

*GPSC, global pneumococcal sequence cluster; IPD, invasive pneumococcal disease; ND, not detected; PBP, penicillin-binding protein; PCV, pneumococcal conjugate vaccine; ST, sequence type.

Comparing of the strains causing IPD in children and older adults in the PCV10 period indicated the same predominant STs (ST416 and ST994). Two STs, ST199 and ST276, accounted for 14.3% (8/56) of 19A strains in older adults; we did not detect those STs in the IPD cases in young children. Conversely, 4 STs we detected in young children who had IPD were not found in older adults.

Serotype 19A strains from the PCV10 period grouped into 9 GPSCs. Strains that had the same ST always grouped together into 1 GPSC. A total of 6 GPSCs consisted of different STs. A total of 63.3% (105/166) of the strains grouped in GPSC4, and 18.1% (30/166) grouped in GPSC146. The other GPSC accounted for <5% of the strains.

### Genomic Characterization of Predominant MLST Types during 2017–2018

ST416 strains are mainly pilus 1 gene positive (76/79), and all are penicillin susceptible, based on the 0-0-0 PBP profile in 74 of the 79 strains. A total of 20.2% (16/79) had the *erm*(B) gene, and all those strains were erythromycin resistant. In 20.2% of ST416 strains, we detected the *tet*(M) gene, conferring phenotypically tetracycline resistance in these strains. ST416 is part of clonal complex 199 and is a double-locus variant of the worldwide distributed penicillin nonsusceptible ST199. According to the pubMLST database (https://pubmlst.org), ST416 has been associated with serotype 19A and to a lesser extend with serotype 19F (only 3 strains in the database as of May 14, 2021). ST416 strains belonged to GPSC4, which is the GPSC with the highest number of serotype 19A strains in the GPSC database (Pathogenwatch, https://www.pneumogen.net; 25,731 pneumococcal genomes as of May 14, 2021). The ST994 strains from Belgium clustered in GPSC146, which is a small GPSC with only 23 public genomes assigned, all ST994 strains.

Of the other predominant 19A strains, ST994 and ST2081 did not have pilus genes and had no erythromycin or tetracycline resistance genes detected; they were phenotypical susceptible to all tested antimicrobial drugs. ST994 has been identified in only 77 isolates, all serotype 19A except 1 serotype 19C isolate, which indicates that it is even less frequently described than ST416 (179 isolates) in the pubMLST database. It is unlikely that this ST results from a serotype switch.

Conversely, ST320 strains carried the 13-11-16 PBP profile associated with penicillin resistance. We also detected *erm*(B), *mef*(A), and *tet*(M) genes in all ST320 strains, which correlated with the phenotypical resistance to erythromycin and tetracycline.

### Comparing Pre-PCV10 to PCV10 Serotype 19A Clones

Comparing serotype 19A strains causing IPD in children in the PCV7 period (2010) and PCV13 period (2012–2014) to those from the PCV10 period (2017–2018) indicated a change in predominant STs and GPSCs over time (Table 3; Figure). The predominant clones in the PCV10 period, ST416 (GPSC4) and ST994 (GPSC146), which accounted for 75.9% (41/54) of serotype 19A IPD strains during 2017–2018, were also detected in the PCV7 and PCV13 period but at much lower proportions: 11.9% (8/67) for the PCV period and 30.4% (7/23) for the PCV13 period. Of the clones that were predominant during the PCV7 period, ST193 (GPSC11) and ST276 (GPSC10) (together 40/67; 59.7%), only 1 ST193 (GPSC11) isolate was detected as the cause of IPD in the youngest children during the PCV10 period. ST416, ST994, ST193 and ST276 clustered in 4 different GPSC, indicating they were not closely related to each other.

**Table 3:** GPSC and ST assignment for serotype 19A invasive pneumococci isolated during PCV7 period (2010), PCV13 period (2012–2014), and PCV10 period (2017–2018), from children <2 years of age, Belgium*

GPSC	Sequence type	No. isolated			
		PCV7 period, 2010	PCV13 period, 2012–2014	PCV10 period, 2017–2018	Total
GPSC4	Total	7	4	28	39
	ST199	2	0	0	2
	ST416	2	4	26	32
	ST876	1	0	0	1
	ST3012	2	0	0	2
	ST3017	0	0	1	1
	ST15414	0	0	1	1
GPSC11	Total	31	5	1	37
	ST193	26	5	1	32
	ST1228	1	0	0	1
	ST2927	2	0	0	2
	ST16995	1	0	0	1
	ST16996	1	0	0	1
GPSC10	Total	20	6	3	29
	ST276	14	6	0	20
	ST2013	4	0	1	5
	ST15077	0	0	2	2
	ST16994	2	0	0	2
GPSC146	ST994	6	3	15	24
GPSC1	Total	1	1	3	5
	ST320	1	1	2	4
	ST9387	0	0	1	1
GPSC9	ST63	1	1	1	3
GPSC18	Total	0	2	0	3
	ST4831	0	1	0	1
	ST99	0	0	0	1
	ST1848	0	1	0	1
GPSC109	ST13096	0	0	2	2
Unknown	Total	1	1	1	1
Total	Total	67	23	54	143

*GPSC, global pneumococcal sequence cluster; PCV, pneumococcal conjugate vaccine; ST, sequence type.

**Figure:**
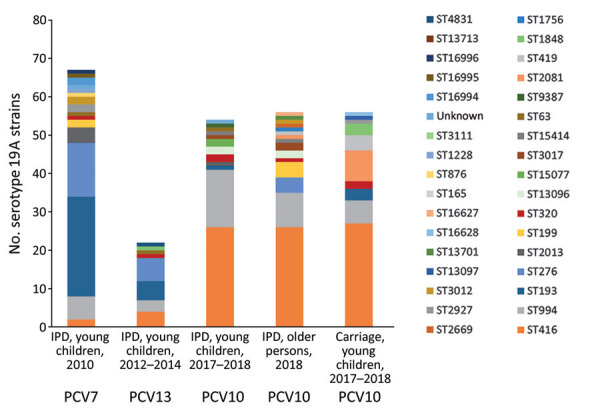
Number and ST distribution of pneumococcal serotype 19A strains isolated from invasive disease and carriage, Belgium. Shown are IPD cases in young children during 2010, 2012–2014, and 2017–2018; serotype 19A strains isolated from IPD cases in older persons during 2018; and serotype 19A strains carried by children during 2017–2018. Different colors indicate different STs. IPD, invasive pneumococcal disease; PCV, pneumococcal conjugate vaccine; ST, sequence type.

## Discussion

After the PCV13 to PCV10 switch in Belgium, a rapid emergence of serotype 19A occurred in IPD and nasopharyngeal carriage in the youngest children. Emergence in both groups was associated with the increase in a variety of mainly penicillin-susceptible serotype 19A clones. Two serotype 19A clones, ST416 (GPSC4) and ST994 (GPSC146), accounted for most 19A isolates. Those clones were not only predominant in the youngest children but were also the predominant clones causing IPD in adults after vaccine switch. These emerging clones differed from the serotype 19A clones (ST193 and ST276) that were mainly responsible for the increase in serotype 19A IPD in children after PCV7 introduction in Belgium and other countries in Europe (*17*–*20*).

The 2 predominant STs are greatly involved in carriage in the youngest children, as well as in invasive disease in the youngest children and older adults, which might indicate that these strains have an advantage to spread compared with other STs of serotype 19A. Although ST416 and ST994 were already on the increase before the vaccine switch in Belgium, they were not the predominant serotype 19A STs in childhood IPD at that time. Instead, ST193 and ST276 still accounted for most of the serotype 19A strains causing IPD in the youngest children during the PCV7 and PCV13 periods. The number of ST193 and ST276 serotype 19A IPD strains in young children did not increase after the vaccine switch. We observed that after introduction of PCV7 and PCV10, an increase in serotype 19A was detected, but the effect on the microepidemiology of serotype 19A was different because of emergence of different clones.

Although serotype 19A is well known for its high level of antimicrobial drug resistance, emergence of serotype 19A after introduction of PCV10 in Belgium is driven mainly by drug-susceptible pneumococci. Although the penicillin resistance rate is somewhat higher in serotype 19A IPD strains (13.0% in adults and 11.1% in young children) compared with carriage strains (5.4%), those resistance rates are much lower than the resistance rates for serotype 19A IPD strains isolated from children before the PCV13 period (e.g., 38.6% in 2011; data from the Belgian Reference Centre for Invasive *S. pneumoniae*).

An influencing factor could be different antimicrobial drug pressure during the PCV7 period in comparison with the PCV10 period. In the PCV10 period (2015–2018; 23.4 defined daily doses [DDDs]**/**1,000 inhabitants/day) a slightly lower consumption of systemic antimicrobial drugs in community and hospital settings was detected than during the PCV7 period (2007–2010; 23.9 DDDs/1,000 inhabitants/day) (*21*). In addition, during 2007‒2015, macrolide consumption gradually increased (2.7 to 3.7 DDDs/1,000 inhabitants/day), and nonpenicillin β-lactam (code J01D in the World Health Organization ATC classification system, https://www.whocc.no/atc_ddd_index; 2.73 to 1.8 DDDs/1,000 inhabitants/day) consumption gradually decreased (*21*). However, because ST416 and ST994 strains are mainly macrolide susceptible, it is unlikely that the modest increase in macrolide antimicrobial drug pressure is the driver of the spread of these specific clones.

Looking into more detail for ST416 and ST994 is useful to clarify why these STs expanded in comparison with other serotype 19A STs. In contrast to the ancestral ST199, ST416 strains were all penicillin susceptible in this study. Some diversity was observed regarding macrolide and tetracycline resistance within the emerging ST416 strains in Belgium, but the resistant strains were seen in equal proportions in the 3 studied groups (IPD children, IPD adults, and carriage children). ST416 has been detected in countries in Europe at relatively low frequencies. In Germany, France, Spain, and Finland, it has been detected after PCV introduction, but it was only responsible for <10% of serotype 19A strains (*17*–*20*). Also in Belgium, ST416 has sporadically been detected before the PCV13 to PCV10 vaccine switch. An exception is Italy, where ST416 accounted for >60% of serotype 19A IPD strains and was the driver of the increase of serotype 19A IPD after introduction of PCV7 (*17*). These data for Italy underscores the potential of ST416 to rapidly increase.

Before 2015, ST994 was sporadically detected in Belgium and in other countries in Europe (e.g., Germany, Spain, and the Netherlands). ST994 was also the predominant 19A clone in children <5 years old after PCV10 introduction in Finland.

A detailed comparison of 19A strains from Belgium with 19A strains from countries using PCV13 and PCV10 is needed to investigate a correlation between presence of ST994 and use of PCV10. Publicly available databases do not contain genome sequences with relevant metadata for ST994 and ST416 serotype 19A strains to investigate whether the serotype 19A from Belgium is distinct from the serotype 19A strains from other countries in Europe using PCV13 or PCV10. To study this possibility in detail, collaboration between national reference centers is needed.

Pilus genes are major virulence factors promoting adhesion, invasion, and spreading of the pneumococcus in the human host. Almost all ST416 strains carry the pilus 1 gene, which might be a competitive advantage for this clone. In general, <40% percent of all invasive pneumococci carry pilus genes, and the presence of pilus genes has been associated with serotype 19A. Presence of pili is frequently associated with antimicrobial drug resistance in pneumococcal strains. In contrast, in this study, pilus genes were detected in penicillin-susceptible strains. The emerging ST416 accounts for most of these pilus 1–positive strains. ST416 and ST994 could also carry other virulence factors, which could explain their competitive advantage compared with other STs.

Only some serotype 19A clones are detected in carriage but not in IPD and vice versa. Children act as a reservoir for the pneumococci that cause invasive disease in older adults, but the dynamics between pneumococcal carriage in young children and IPD in the same age group and older age groups are not yet fully understood (*22*–*24*). In this study, the same predominant clones of serotype 19A are responsible for the emergence of serotype 19A, indicating an association between strains that are carried by young children and strains that cause invasive disease in children and adults. Other invasive serotypes (e.g., serotype 1, 8, and 12F) are not frequently carried in the youngest children, which suggests that also other factors are essential for the spread of pneumococci that cause invasive disease.

Based on the increase of total IPD and serotype 19A IPD in the youngest children in Belgium, PCV10 was again replaced by PCV13 in September 2019 (*25*,*26*). Data from Belgium for emerging serotype 19A clones in adults and children are useful for other countries that switched from PCV13 to PCV10 or that plan to make changes on the dose schedule and type of vaccine. Close monitoring of IPD epidemiology by surveillance is needed to rapidly detect emerging clones. Investigation of the microepidemiology of serotype 19A after this switch back from PCV10 to PCV13 will be useful for further investigation of correlations between the use of the different PCVs and the circulation of specific serotype 19A clones. However, confounding variables caused by the coronavirus disease pandemic and its related containment measures during 2020 and 2021, which resulted in a perturbation of the IPD epidemiology in Belgium and other countries, will make this analysis more complicated (*27*,*28*).
